# Does V1 response suppression initiate binocular rivalry?

**DOI:** 10.1016/j.isci.2023.107359

**Published:** 2023-07-08

**Authors:** Brock M. Carlson, Blake A. Mitchell, Kacie Dougherty, Jacob A. Westerberg, Michele A. Cox, Alexander Maier

**Affiliations:** 1 Department of Psychology, College of Arts and Science, Vanderbilt Vision Research Center, Center for Integrative and Cognitive Neuroscience, Vanderbilt University, Nashville, TN 37235, USA; 2 Department of Psychology, Princeton Neuroscience Institute, Princeton University, Princeton, NJ 08540, USA; 3 Department of Vision and Cognition, Netherlands Institute for Neuroscience, Royal Netherlands Academy of Arts and Sciences, Amsterdam 1105 BA, the Netherlands; 4 Center for Visual Science, University of Rochester, Rochester, NY 14627, USA

**Keywords:** Cellular neuroscience, Sensory neuroscience

## Abstract

During binocular rivalry (BR) only one eye’s view is perceived. Neural underpinnings of BR are debated. Recent studies suggest that primary visual cortex (V1) initiates BR. One trigger might be response suppression across most V1 neurons at the onset of BR. Here, we utilize a variant of BR called binocular rivalry flash suppression (BRFS) to test this hypothesis. BRFS is identical to BR, except stimuli are shown with a ∼1s delay. If V1 response suppression was required to initiate BR, it should occur during BRFS as well. To test this, we compared V1 spiking in two macaques observing BRFS. We found that BRFS resulted in response facilitation rather than response suppression across V1 neurons. However, BRFS still reduces responses in a subset of V1 neurons due to the adaptive effects of asynchronous stimulus presentation. We argue that this selective response suppression could serve as an alternate initiator of BR.

## Highlights

• The role of primary visual cortex (V1) for binocular rivalry (BR) is unclear
• V1 population spiking is reduced at the onset of BR, providing a potential trigger
• However, this broad spiking suppression does not occur for a variant of BR
• The BR variant reduces subpopulation responses, a potential alternate trigger

## Graphical abstract

**Figure:**
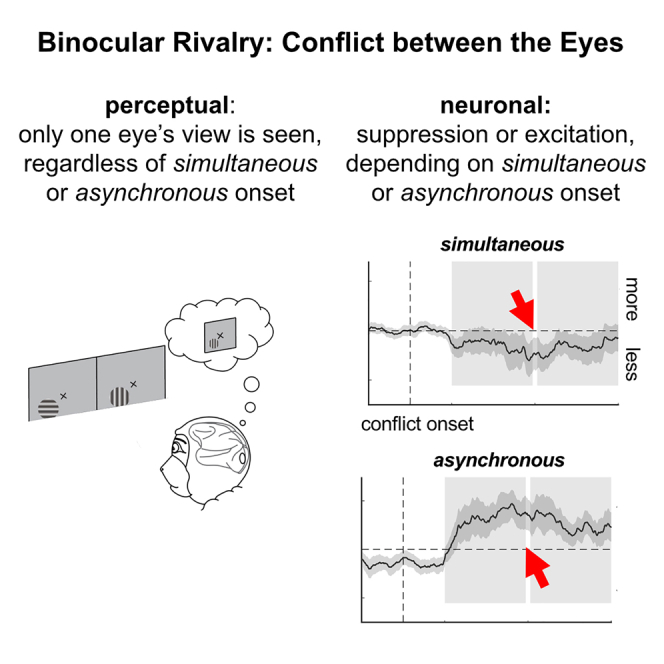

## Introduction

Seeing something different in each eye (dichoptic viewing) is a constant part of the visual experience. One reason that the two eyes’ views are not identical is that the horizontal offset between the eyes causes a geometrical difference in perspective.^1^ Each eye’s view is also distinct because of differences in the obstruction by both the nose and the retinal blind spots.^2^ Furthermore, the two peripheral visual fields can only be seen by either eye.^3^

One of the main roles of the primary visual cortex (V1) is to resolve minor differences between each eye’s view to produce fused (“cyclopean”) visual information to feedforward to the rest of the visual system.^4^^,^^5^^,^^6^ However, larger differences between the two eyes’ views cannot be reconciled. In this case, our visual system either resorts to double vision (diplopia)^7^^,^^8^ or one eye’s view dominates over the other (binocular rivalry).^9^^,^^10^^,^^11^^,^^12^^,^^13^^,^^14^^,^^15^^,^^16^^,^^17^^,^^18^^,^^19^^,^^20^^,^^21^^,^^22^

Binocular rivalry is a fascinating phenomenon with wide-ranging implications, from neural mechanisms of binocular vision^23^^,^^24^^,^^25^^,^^26^ to the neural correlates of consciousness.^11^^,^^17^^,^^27^^,^^28^^,^^29^^,^^30^^,^^31^ As a consequence, binocular rivalry has been studied extensively both on the psychophysical as well as on the neuronal level.^9^^,^^14^^,^^32^^,^^33^^,^^34^^,^^35^ Despite the large body of work on binocular rivalry, there are still open questions about its neural basis.

Increasing proportions of neurons correlate with perceptual reports of binocular rivalry as the hierarchy of cortical visual areas is ascended.^14^^,^^31^^,^^36^^,^^37^^,^^38^^,^^39^^,^^40^^,^^41^^,^^42^^,^^43^^,^^44^^,^^45^^,^^46^ Notably, initial studies found that less than a quarter of neurons in V1 modulate with perception during rivalry.^42^ In contrast, neuroimaging (fMRI) indicates that V1 modulates strongly with the perceptual state of the subject.^47^^,^^48^ This finding holds even within subjects, and it is still unclear what causes the apparent discrepancy^49^ Taken together, these findings suggest that neural responses associated with binocular rivalry occur across the entire visual system,^2^^,^^9^^,^^21^ including – to some degree – V1. However, the precise role that V1 plays in this intricate network of rivalry-related activation is still unclear. It remains uncertain if V1 predominantly contributes to the detection of interocular conflict, the suppression of one eye’s view, or the initiation of reversal between each eye’s perspective.

One intriguing idea is that V1 may be needed to *initiate* rivalry. This concept has gained special importance as discrete stages of binocular rivalry – particularly onset rivalry – have been identified.^50^^,^^51^^,^^52^^,^^53^ Furthermore, several computational models^54^^,^^55^^,^^56^^,^^57^^,^^58^^,^^59^^,^^60^ and V1 neurophysiology results^61^^,^^62^^,^^63^^,^^64^^,^^65^ (see next paragraph) have suggested that V1 may initiate rivalry through mechanisms of neural inhibition. Most importantly, fMRI has revealed neural signatures that indicate ignition of rivalry in V1.^47^ This evidence, taken together, suggests that V1 acts as a key locus of binocular rivalry initiation.^30^^,^^66^^,^^67^^,^^68^ On this view, V1 is crucially involved in binocular rivalry, even though the perceptual phenomenon of seeing one eye’s view arises at an attention-dependent stage of processing outside V1^69^^,^^70^^,^^71^ (but see Watanabe et al.^72^).

If V1 were to play a crucial role for the initiation of binocular rivalry, how could this be conducted at the cellular level? Several computational models propose that response suppression allows for instability in neural population responses, thus enabling the initiation of rivalry.^56^^,^^73^^,^^74^^,^^75^ A specific mechanism of V1 response suppression that could act as initiator of binocular rivalry is dichoptic cross-orientation suppression (dCOS).^61^^,^^62^^,^^63^^,^^64^^,^^65^ dCOS refers to an unspecific reduction of V1 responses that occurs during conflicting binocular stimulation.^64^ In other words, onset of binocular rivalry-inducing stimuli reduces V1 spiking responses across all types of V1 neurons, no matter their specific tuning properties or the perceptual state of the subject^61^^,^^62^^,^^63^^,^^64^^,^^65^^,^^76^^,^^77^ (and see DeAngelis et al.^78^). For ease of communication, we will refer to this phenomenon as response suppression (to dichoptic stimuli).

One reason that response suppression to dichoptic stimulation is interesting is that the onset of response suppression roughly coincides with the perceptual onset of binocular rivalry.^52^^,^^53^^,^^61^^,^^63^ This observation can be seen as further evidence that the resulting weak neural response might contribute to an overall destabilization of the cortical representation of stimuli paving the way for binocular rivalry’s characteristic perceptual instability.

One way to evaluate this hypothesis is to investigate whether neural suppression is a precursor to *all* forms of binocular rivalry. If neural suppression does not occur under every circumstance of dichoptic stimulation that evokes binocular rivalry, it is unlikely to serve as a prerequisite for binocular rivalry. Here, we use binocular rivalry flash suppression (BRFS) to assess whether the ignition of binocular rivalry requires neural suppression at the moment of dichoptic onset. BRFS is a particular form of binocular rivalry where one eye is adapted before the onset of dichoptic stimulation.^79^ This monocular adaptation period forces the secondarily presented – or *flashed stimulus* – to dominate perception for ∼1 s.^70^^,^^79^^,^^80^ Importantly, the psychophysical properties of BRFS closely resemble those of binocular rivalry, suggesting that these two phenomena share a neural mechanism^29^^,^^41^^,^^73^^,^^79^^,^^81^^,^^82^^,^^83^ (and see Ooi & Loop^84^).

Our study shows that primate V1 neurons do not undergo unspecific response suppression at the onset of binocular rivalry when using BRFS. Instead, we observed transient response enhancement (facilitation). This outcome challenges the notion that response suppression is needed for initiation of binocular rivalry. We propose that the reduction of V1 activity due to adaptation may serve a similar role during BRFS. That is, adaptation affects both eye-specific and stimulus-specific V1 neural populations. This eye-specificity and stimulus-specificity of reduced responses may be one reason BRFS leads to a more deterministic perceptual outcome compared to regular binocular rivalry.

## Results

**Figure 1:**
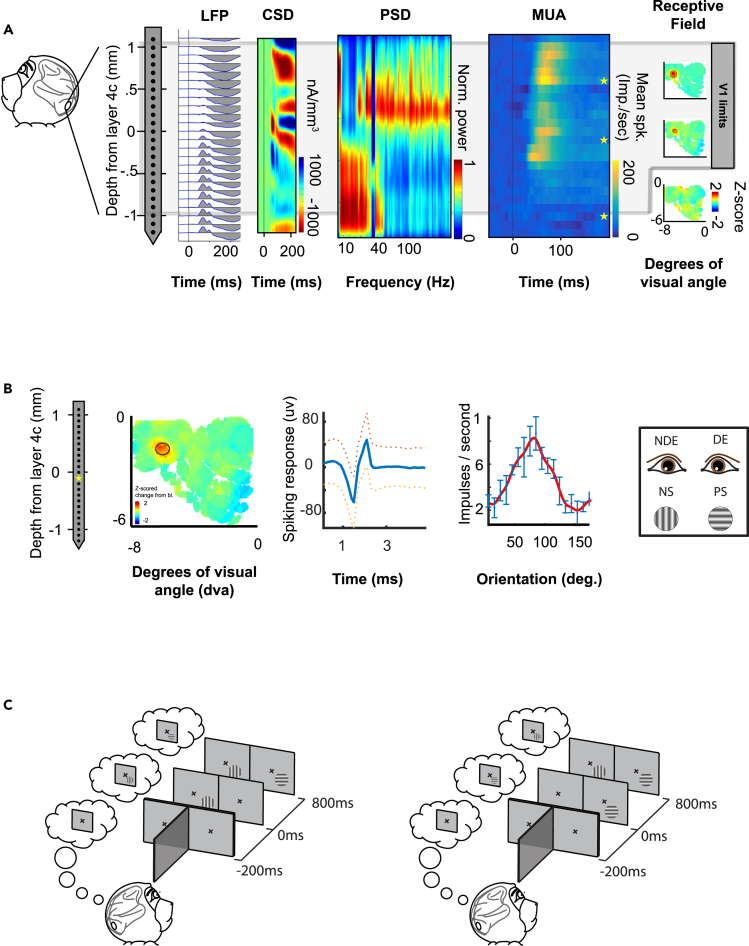
Binocular rivalry flash suppression (BRFS) paradigm (A) Linear multielectrode arrays recorded neurophysiology data from primary visual cortex (V1) of two awake and behaving macaques (*Macaca radiata*). The cortical boundaries of V1 were determined with convergent results from local field potentials (LFP), current source density (CSD), power spectral density (PSD), mean multi-unit activity (MUA), and receptive field mapping (see STAR Methods). The granular input layer (4c) is the main reference point observed in LFP, CSD, and PSD. As such, layer 4c is set to a depth of 0mm, and the rest of the cortical depth is referenced from its lower laminar boundary. The electrode corresponding to -1mm below layer 4c does demonstrate significant responses to any receptive field, indicating that it is placed in subcortical white matter. (B) Eye preference and orientation tuning were determined for each V1 multi-unit. To be included in our analysis multi-units had to exhibit statistically significant response differences for eye stimulation and stimulus orientation. (C) Subjects sat in front of a mirror stereoscope that allowed for monocular stimulation while the subject maintained binocular fusion. The subjects’ task was to maintain fixation within a 1-degree window until the end of stimulus presentation. During BRFS trials, monocular adaptation for 800ms causes perception to switch to the unadapted eye when binocular rivalry begins at the onset of dichoptic presentation. Thought bubbles indicate the fused (cyclopean) percept of the subject.

**Figure 2:**
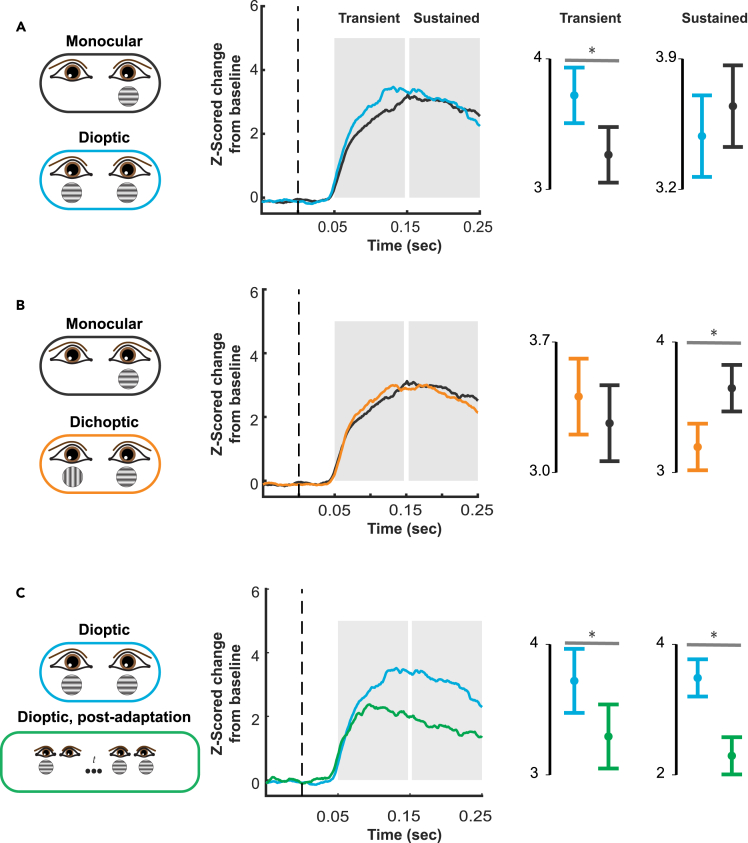
Control conditions (A) Monocular versus dioptic stimulation. Solid lines represent the grand average multi-unit responses for 91 significantly tuned V1 units (across both animals). Spiking responses are normalized per unit and represented as z-scored change from baseline. Shaded areas represent windows for the transient (50–150ms) and sustained (150–250ms) responses. Mean responses rates for these windows are shown in the far-right column. Asterisks indicate significance at α = 0.05 using Wilcoxon signed-rank tests. Black: Monocular presentation of preferred orientation to the neurons’ preferred (dominant) eye. Blue: Dioptic presentation of preferred stimuli. (B) Monocular versus dichoptic stimulation. Responses are grand averages of the same units as in A. Black: Monocular presentation of preferred stimuli to dominant eye. Orange: Dichoptic presentation of the preferred stimulus in the dominant eye and the null stimulus in the non-dominant eye. Note the response suppression for dichoptic stimulation in the sustained response window. (C) Simultaneous versus asynchronous stimulation. Blue: Dioptic presentation of preferred stimuli. Green: Dioptic presentation of preferred stimuli following monocular adaptation of a preferred stimulus in the non-dominant eye.

Our aim was to test the hypothesis that response suppression as a result of dichoptic stimulation functions as a prerequisite step for the initiation of binocular rivalry. To do this, we evaluated whether V1 spiking responses to BRFS diminished at the onset of interocular conflict (rivalrous stimulation) (Figure 1). As a control, we first aimed to reproduce the existence of response suppression for conventional binocular rivalry that occurs following *simultaneous* onset of *dichoptic* gratings (Figures 2A and 2B). This *dichoptic* control allowed us to quantify the impact of interocular conflict on V1 spiking. Secondarily, we examined the influence of adaptation due to *asynchronous* onset on V1 responses during BRFS, because it is stimulus onset asynchrony (SOA) that differentiates BRFS from conventional binocular rivalry (Figure 2C). To do so, we replicated the BRFS stimulus sequence, but presented a stimulus to the unadapted eye that was identical to (congruent with) the monocular adapter. This *dioptic* control allowed us to quantify the effects of BRFS-related adaptation. As such, we were able to look for independent (dissociated) influences from *adaptation* and *dichoptic* conflict on V1 response rates.

### Response suppression follows the onset of dichoptic stimulation

Prior studies on conventional binocular rivalry – with simultaneous onset of each eye’s stimulus – found a reduction in V1 spiking responses. This response suppression occurred independently of the neurons’ selectivity for stimulus orientation or the perceptual state of the subject (“interocular suppression” or “dichoptic cross-orientation suppression”).^61^^,^^62^^,^^63^^,^^64^^,^^65^^,^^76^^,^^85^^,^^86^^,^^87^^,^^88^^,^^89^ The response suppression from dichoptic stimulation is typically observed 150–250ms after stimulus onset, which temporally matches the perceptual onset of binocular rivalry.^3^^,^^9^^,^^61^^,^^73^^,^^90^

For full evaluation, we compared the responses of monocular stimulation (black) to both dichoptic (red) and dioptic stimulation (blue). Note that a preferred and non-preferred stimulus were chosen for each acute penetration based on columnar population responses. Therefore, not every multi-unit on a given penetration had perfectly matching orientation and eye selectivity. We thus limited our statistical analyses to 91 (out of 219 tuned) V1 multi units that exhibited significant response preferences that aligned with our stimuli. Pairwise comparisons between conditions were evaluated for what we defined as both the transient (50–150ms) and sustained (150–250ms) response periods.

Dioptic stimulation initially resulted in higher multi-unit activity (MUA) responses than monocular stimulation during the transient phase of the visually evoked spiking response (Figure 2A). Responses during the transient phase did not indicate a deviation from normality under a Shapiro–Wilk test (W = 0.985, p = 0.840). As such, a paired-samples Student’s *t* test was used, and indicated that this difference was statistically significant, (t = 3.051, p = 0.004, 95% CI [0.154, 0.757], Cohen’s d = 0.460). During the sustained phase, the difference between dioptic and monocular conditions was not statistically significant, indicating a form of response normalization (Shapiro–Wilk: W = 0.960, p = 0.132; Student’s *t* = −1.047, p = 0.301, 95% CI [-0.470, 0.149]) (see Mitchell et al.^26^ and Cox et al.^61^ for further work on this subject).

Previous studies evaluating the suppression of neural responses as a result of dichoptic stimulation used a variety of stimulus contrast levels.^64^^,^^65^^,^^77^^,^^88^^,^^91^^,^^92^ Here, we settled on medium-high contrast, balanced across the eyes (see STAR Methods). In order to rule out that our choice of contrast confounded the presence of response suppression during BRFS, we first tested for response suppression using our stimulus set. In other words, we first exposed the animals to regular binocular rivalry, where stimuli are presented simultaneously (Figure 2B) to replicate the classic observation of response suppression as a result of dichoptic stimulation. To do so, we simultaneously presented two dichoptic gratings at the same location of visual space, one in each eye (see STAR Methods for details). These dichoptic gratings had orthogonal orientations.^65^^,^^77^

The initial transient response rate did not statistically differ between dichoptic stimulation and monocular stimulation. Since a Shapiro-Wilk test of normality suggested deviation from normality (W = 0.948, p = 0.046), a Wilcoxon signed-rank test was used for the paired samples t-test (W = 514, z = 0.495, p = 0.625). The Hodges-Lehmann estimate was used for the 95% confidence interval ([-0.214, 0.377]). This analysis revealed response suppression during the sustained phase of the response (here defined as 150–250ms). That is, dichoptic stimulation resulted in significantly lower response rates compared to monocular stimulation. Here, too, a Shapiro–Wilk test indicated deviation from normality (W = 0.919, p = 0.004), so a Wilcoxon signed-rank test was used for the paired samples t-test (W = 212, z = −2.995, p = 0.003), and the Hodges–Lehmann estimate was used for the 95% confidence interval ([-0.698, −0.121]). Medium effect size is given by the matched rank biserial correlation (MRBC = −0.53). While constituting a replication of earlier findings, this response suppression is remarkable. It amounts to a single eye’s stimulation eliciting *more* V1 excitation than stimulating both eyes simultaneously, despite retaining the same stimulus in the same eye.

### Monocular adaptation greatly reduces the initial transient of binocular responses

We next revisited how V1 neurons respond to (monocular) adaptation. It is well established that adaptation elicits profound reduction of V1 responses.^93^^,^^94^^,^^95^^,^^96^^,^^97^^,^^98^^,^^99^^,^^100^^,^^101^^,^^102^^,^^103^^,^^104^^,^^105^^,^^106^^,^^107^^,^^108^^,^^109^^,^^110^^,^^111^^,^^112^^,^^113^^,^^114^^,^^115^^,^^116^ In this specific case, we were interested in how far adaptation to one eye affects V1 responses to stimuli shown to both eyes.

Figure 2C shows the effect of monocular adaptation on subsequent dioptic stimulation (i.e., how exposing one eye to a stimulus affects responses to adding the same stimulus to the other eye). This is an essential control for BRFS, as it has the same temporal and ocular stimulation pattern, *modulo* dichoptic conflict. As a result, this stimulation sequence causes perceptual fusion rather than binocular rivalry. Adaptation was always restricted to the neurons’ non-dominant eye. After 800ms stimulus onset asynchrony, the second stimulus was shown to the dominant eye while the (adaptor) stimulus in the non-dominant eye remained on screen.

We found that monocular adaptation significantly reduced binocular neural responses during both the initial transient and sustained response windows. A Shapiro–Wilk test of normality did not indicate that results deviated from normality during the transient phase (W = 0.967, p = 0.234), so a Student’s *t* test was used (t = 2.477, p = 0.017, 90% CI [0.z079, 0.773], Cohen’s d = 0.373). The sustained phase responses deviated from normality (W = 0.892, p < 0.001). Therefore, non-parametric tests were used for evaluating the later (W = 935, z = 5.135, p < 0.001, 95% CI for Hodges–Lehmann estimate [0.697, 1.481], MRBC = 0.889). To sum, monocular adaptation significantly reduces V1 responses to the onset of a *dioptic* stimulus (consisting of two copies of the adapter). It seems reasonable to assume that at least part of this response reduction also carries over to the situation where binocular stimulation consists of one copy of the adapter, paired with a novel stimulus (as is the case for BRFS). Since these results confirm that both (1) monocular adaptation reduces V1 responses and (2) dichoptic stimulation reduces V1 responses, we expected V1 responses to show a clear reduction at the onset of the dichoptic stimuli following monocular adaptation during BRFS.

### Facilitation, not suppression, follows onset of BRFS

**Figure 3:**
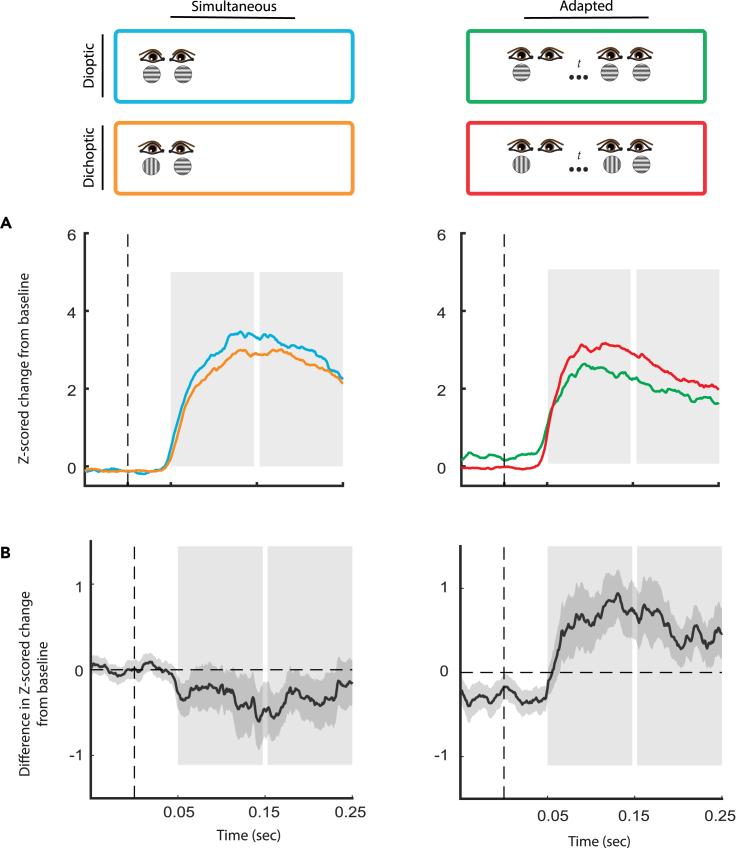
Response suppression is not observed during BRFS (A) Solid lines: Grand average V1 responses (N = 91). Pairwise comparisons between dioptic and dichoptic stimulus presentations for simultaneous presentations (left column) versus asynchronous presentations (right column). Blue: simultaneous dioptic stimulation. Orange: simultaneous dichoptic stimulation. Red: dioptic stimulation following monocular adaptation. Green: BRFS, consisting of dichoptic stimulation following monocular adaptation. Note that the preferred stimulus is always shown to the dominant eye across conditions. (B) Average difference in V1 neural response between dioptic and dichoptic conditions. Negative values indicate response suppression and positive values indicate response facilitation.

As our final step, we investigated whether BRFS results in response suppression. To find out, we contrasted the onset of dichoptic stimulation during BRFS (light red in Figure 3) with the onset of dioptic stimulation following monocular adaptation (light blue in Figure 3). Prior work often described dichoptic response suppression with respect to monocular stimulation.^61^^,^^63^^,^^65^ However, we chose to compare dichoptic to dioptic stimulation to account for the substantial neural suppression induced by monocular adaptation discussed above. This shift in definition can be justified by the observation that sustained responses do not deviate between dioptic and monocular stimulation (Figure 2A). In line with our revised definition, prior literature has consistently noted that dichoptic stimulation not only results in reduced responses when compared to monocular stimulation, but also when compared to dioptic stimulation.^61^^,^^63^^,^^64^^,^^65^^,^^77^^,^^117^

We first tested for decreased neural responses during the sustained response (150–250ms), where dichoptic response suppression was previously reported.^61^ Note that this response period roughly coincides with a distinct phase during binocular rivalry onset where initial fusion subsides.^50^^,^^51^^,^^52^^,^^118^ However, dichoptic (BRFS) responses were not significantly different from their dioptic counterpart during this time, as indicated by a Wilcoxon signed-rank test (T = 523, z = −1.105, p = 0.271, 95% CI [-0.363, 0.102]). In other words, response suppression was not observed within the context of BRFS.

Further investigation revealed that there was a significant response difference during the initial response transient. However, this difference was due to *increased* (facilitated) responses during BRFS. This difference was significant, as indicated by a Wilcoxon signed-rank test (T = 81, z = −5.455, p < 0.001, 95% CI [-1.724, −0.842], MRBC = −0.878, 95% CI of MRBC [-0.933, −0.782]). This means that following adaptation, showing a neuron’s preferred stimulus in duplicate – one to each eye – results in a *smaller* response than showing it once, paired with a non-preferred stimulus in the other eye.

### Dichoptic facilitation during BRFS is consistent across varying levels of excitatory drive

**Figure 4:**
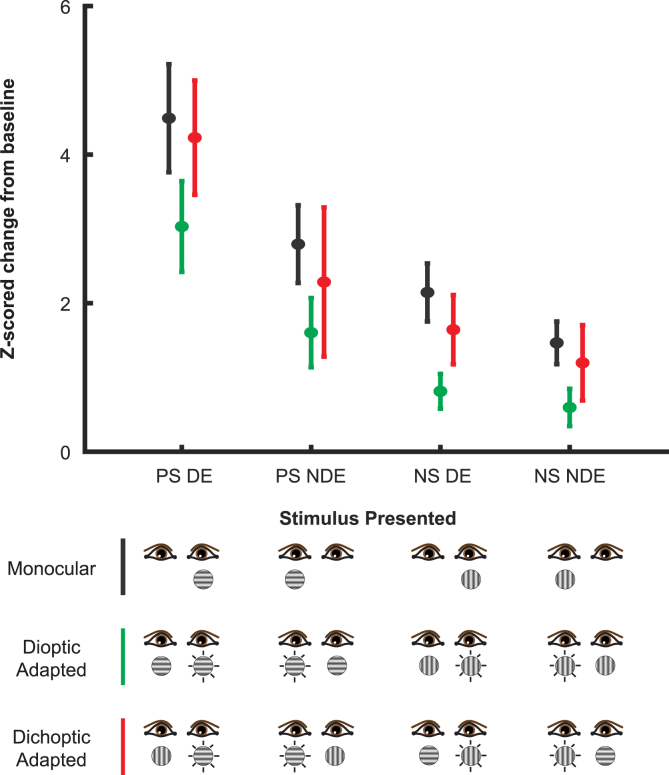
Response facilitation during BRFS is independent of stimulation context Response means (50-150ms post stimulus onset) and 95% confidence intervals (N = 91) for monocular presentations (black), asynchronous dioptic presentations following monocular adaptation (green), and BRFS (red). The preferred stimulus (PS) and dominant eye (DE) evoke greater responses (“drive”) than the null stimulus (NS) and the non-dominant eye (NDE). Note that no matter the amount drive, BRFS always evokes a larger response than the asynchronous dioptic control condition.

So far, our considerations were limited to a specific combination of stimulus orientation and ocular configuration (i.e., preferred stimulus in dominant eye). We next widened our analyses to other combinatorial possibilities, as seen in Figure 4. This is an important comparison since it can help account for neural “drive”. The concept of drive is equivalent to stimulus properties that enhance responses. Maximal drive was elicited by presenting the preferred stimulus orientation in a neuron’s preferred (dominant) eye. Minimal drive, in turn, was elicited by showing the null stimulus to the non-dominant eye. Intermediate drive was elicited by the remaining combinations (i.e., preferred orientation in non-preferred eye and null orientation in preferred eye). These comparisons were evaluated over the transient response period (50-150ms) (Figure 3).

Across these varying levels of drive, the lowest response was consistently evoked by dioptic presentations following monocular adaptation (Figure 4). A two-way repeated measures ANOVA was performed across the transient phase of all responses. The first factor is visualized as the black, blue, and red-indicated conditions in Figure 4. The second factor consisted of the stimulus type: preferred stimulus, non-preferred stimulus, preferred eye, and non-dominant eye. This factor is visualized as the groupings along the abscissa of Figure 4. Mauchly’s test of sphericity indicates that the assumption of sphericity is violated (p < 0.05) in all samples, so a Greenhouse–Geisser sphericity correction was used. The two-way repeated measures ANOVA revealed a statistically significant interaction between the effects of condition type and stimulus presentation (F_3.33, 113_ = 4.550, p = 0.003, η^2^ = 0.041) (Tables 1 and 2). One way to interpret the above findings is that BRFS results in stronger responses than its dioptic counterpart since the dioptic control doubles presentation of an adapted stimulus, whereas BRFS introduces a novel, unadapted stimulus. This unadapted stimulus excites a largely unadapted population of V1 neurons.

**Table 1:** Repeated measures two-way ANOVA on transient excitation

Cases	Sum of squares	df	Mean square	F	p	η^2^
*Condition*	24.786	1.383	17.923	8.586	0.002	0.029
Residuals	98.150	47.020	2.087			
*Stimulus*	284.338	1.173	242.367	27.040	<0.001	0.328
Residuals	357.531	39.888	8.963			
*Condition*✻*Stimulus*	12.097	3.333	3.630	4.550	0.003	0.014
Residuals	90.395	113.321	0.798			

Condition stands for the various temporal stimulus types of monocular, dioptic adapted, and dichoptic adapted. Stimulus refers to eye and orientation combination, such as the preferred stimulus presented to the dominant eye, or the null stimulus presented to the non-dominant eye.

## Discussion

BRFS^79^^,^^119^ and its sister phenomena – continuous flash suppression (CFS)^120^ and generalized flash suppression (GFS)^80^ – are all closely related to binocular rivalry. Just like binocular rivalry, flash suppression paradigms utilize discrepant binocular stimulation to evoke suppression of one eye’s view over the other (although there are also instances where interocular conflict is not a requirement).^8^^,^^80^^,^^81^^,^^121^^,^^122^^,^^123^^,^^124^ And, just like binocular rivalry, prolonged stimulation under these paradigms leads to stochastic alternations, or reversals, between each eye’s perspectives.

### The role of primary visual cortex (V1) for binocular rivalry

One of the most widely debated questions about the neural basis of binocular rivalry is the role of V1.^17^^,^^21^^,^^35^^,^^37^^,^^125^^,^^126^^,^^127^^,^^128^^,^^129^^,^^130^^,^^131^ One impetus behind this debate are discrepant results between fMRI and neurophysiological measurements of spiking discharges. While fMRI yields robust V1 signals reflecting the alternating perceptual states of binocular rivalry,^47^^,^^48^^,^^132^^,^^133^^,^^134^ less than a quarter of V1 neurons have been found to mirror this activity^36^^,^^38^^,^^40^^,^^135^^,^^136^ (and see Xu et al.^46^). Assuming a linear, or close to linear, relationship between spiking activity and the fMRI (BOLD) response measured across the same volume of cortex,^137^ results do not seem to converge across these studies.

Initial studies were confounded by the fact that fMRI was used in humans while measurements of spikes were performed in animal species. However, a more recent study showed that fMRI and single neuron spiking measurements in the same individuals still show this discrepancy^49^ (see Anenberg et al.^138^ for a possible mechanistic explanation, and see Watanabe et al.^72^ for a different interpretation). In other words, the answer to whether or not perceptual alternations during rivalry strongly modulate V1 activity depends on the technique that is used to assess this question, not the specific model or paradigm.^31^ To date, it is still unclear what causes this divergence between signals. Several more studies found divergences between fMRI and local neural activity outside binocular rivalry,^139^^,^^140^^,^^141^^,^^142^^,^^143^^,^^144^^,^^145^ suggesting that these two measurements are not as closely linked as previously assumed.

One potential explanation for this dissociation is that fMRI is more sensitive to subthreshold synaptic activation.^146^^,^^147^^,^^148^^,^^149^^,^^150^ This hypothesis is supported by the finding that local field potentials (LFPs), which are believed to arise from (subthreshold) synaptic activity more closely predict the fMRI signal than local (suprathreshold) neuronal spiking.^148^^,^^151^^,^^152^ Indeed, V1 LFP reflects perceptual changes during rivalry, in concert with the fMRI signal, in V1.^49^ Feedback projections from higher visual areas to V1 are one potential source that might produce such subthreshold synaptic activity.^153^ On this view, the V1 feedback modulation observed in fMRI and LFP is not fully translated into V1’s spiking output, and is thus largely epiphenomenal in nature.

Another possibility is that the sampling of V1 neurons using standard *in vivo* neurophysiological techniques is systematically biased toward certain cell types and thus not fully representative of the whole neuronal population. In other words, fMRI might represent a population signal that weighs some types of neurons more heavily than others. There are several plausible possibilities for this to be the case, such as neurons in different layers of V1 differentially affecting the neurovascular coupling that underlies the fMRI signal.^154^^,^^155^^,^^156^^,^^157^^,^^158^ On the flip side, it is also possible that there are different biochemical or morphological classes of neurons that are either under- or oversampled in *in vivo* neurophysiology. Some of the under-sampled types of neurons might hold a disproportionate influence over neurovascular coupling and the hemodynamic response.^159^^,^^160^ In other words, the relationship between the fMRI signal and local spiking may be (highly) non-linear and more complex with respect to specific populations of neurons.^161^^,^^162^

Our study explicitly circumvents these issues by focusing on a simpler question: Does systemic suppression of V1 population activity at the *initiation* of rivalry trigger perceptual suppression of one eye’s view (i.e., binocular rivalry)? Notably, this time window of interest *precedes* perceptual alternations of regular binocular rivalry. We asked whether the starting point for the neural mechanisms that result in perceptual limitation to one eye’s view (perceptual suppression) can be found within V1’s response. In other words, we are interested in the brief moment of onset rivalry,^50^ and the neural ignition that precedes it.

### V1 response suppression as a potential trigger mechanism for initiating perceptual suppression

What evidence speaks of V1 as the initiator for the onset of binocular rivalry? There are at least two lines of reasoning that justify this assumption. First, there is direct evidence derived from fMRI studies of BRFS, Second, the phenomenon of dichoptic (cross orientation) suppression that has been described on the level of single V1 neurons has already been implicated by researchers in the field. We will discuss each of these arguments in succession.

The first line of evidence is based on fMRI studies and stems from a modified BRFS paradigm. The most common set of stimuli for binocular rivalry and BRFS are (Cartesian) orthogonal gratings. Using these stimuli, perceptual dominance and suppression can occur in a piecemeal fashion, consisting of a mosaic of each eye’s view.^2^^,^^163^ Piecemeal rivalry is highly dynamic, giving the impression of fluid changes of the stimulus mosaic. Using non-Cartesian, radial stimuli (i.e., a sunburst pattern that rivals concentric rings), this fluidity becomes more ordered in that the transitions between each eye’s view progress along the lines of the concentric rings. In other words, using these stimuli, perceptual alternations take on the form of traveling waves along a circular path.

Remarkably, this spatiotemporal pattern of a binocular rivalry traveling wave can be traced along V1’s surface using fMRI,^47^ as well as using voltage sensitive dyes in the primate model.^68^ This stimulus can be used for a locally spreading variant of BRFS. If the experimenter enhances the contrast of a small region of the stimulus in the perceptually suppressed eye, this “flashed” region will immediately gain perceptual dominance, just like in regular BRFS. Immediately following this *local* flash suppression, perceptual dominance continues to spread as a traveling wave. In other words, using this local variant of flash suppression, it is possible to trigger binocular rivalry traveling waves in V1, which are traceable using fMRI.

Using the above-mentioned visual paradigm in combination with an attention task yielded a surprising result: When an observer’s attention is allocated to a part of visual space that does not contain interocular conflict, such as a fixation point in the center of the screen, flash-initiated binocular rivalry traveling waves are still observable with fMRI of V1.^132^ And yet, these waves fail to be observed in all downstream visual areas. That is, when observers do not actively attend to the interocular conflict, V1 still initiates binocular rivalry, but the rest of the brain ignores that. One way to interpret this finding is that attention is required to “gate” V1’s rivalry initiation signal to the rest of the visual system.^30^^,^^164^ Indeed, absence of attention seems to severely impact the perceptual outcome of both BRFS^165^ and binocular rivalry.^69^^,^^72^^,^^166^ Another interpretation is that V1 initiates perceptual dominance and suppression, with higher visual areas carrying out the actual task of establishing neural correlates of conscious perception.^167^ This hypothesis of V1 serving as the locus of an initiator for binocular rivalry also rests well with the fact that V1 is the primary locus of binocular combination.^168^^,^^169^

The second line of evidence stems from neurophysiological recordings in cat area A17/A18 (the homolog of primate V1). Recently replicated in macaques, these recordings demonstrated that binocular rivalry-inducing stimuli lead to response suppression.^61^^,^^62^^,^^64^^,^^65^^,^^77^^,^^170^^,^^171^ In theory, this widespread response suppression could serve as a trigger mechanism that allows V1 to prepare the rest of the visual system to induce alternating perceptual dominance and suppression.

One way to conceptualize this hypothesis is from the perspective of *noise-driven attractor models* of binocular rivalry.^75^^,^^172^ A strong binocular response would resemble a stable attractor in the form of a *global* minimum. That is, whenever V1 faces stimuli that can be fused across the eyes, the resulting response is more stable than that of showing a stimulus to one eye only – far away from the random noise fluctuations that occur in the absence of visual stimulation. Such a stable attractor can be conceptualized as a deep cavity, or well, in a flat landscape that a ball rolls into. Once the ball enters such a deep well, it remains in position and the system keeps a steady state (hence: global minimum). When conflict is detected between the eyes, V1 responses are generally suppressed. This weakening of responses can be conceptualized as elimination of a global minimum that guarantees a stable state. Instead, V1’s activity now is closer to (and thus more subject to) random noise fluctuations. As a result, V1’s state is less stable. Using the attractor well metaphor again, we can assume that this means that the ball we envisioned is now not falling into a deep well anymore, where it will rest. Instead, smaller, *local* minima in the form of minute indentations in the flat landscape become new, but less reliable attractor states. That is, the random fluctuations between two semi-stable states (i.e., left eye’s view versus right eye’s view) are akin to two neighboring, small wells between which the system (ball) irregulary fluctuates. Once the ball falls into one of these two local minima, it will remain there for a limited amount of time. A system with constant energy flux (such as a ball remaining in motion) will eventually leave such a local minimum, or attractor state, only to get trapped again by the opposing, shallow well. All the above provides a metaphorical explanation for V1 as a potential locus to initiate binocular rivalry perception, with response suppression as a plausible trigger mechanism.

### Response suppression from interocular conflict does not trigger perceptual suppression during BRFS

Following the above reasoning, we hypothesized that V1 response suppression acts as a trigger for binocular rivalry perceptual suppression. Indeed, our dataset demonstrated response suppression induced by (simultaneous) dichoptic stimulation. Our test of this hypothesis revealed that our prediction does not hold for all cases of binocular rivalry. During BRFS, V1 does not show concurrent neural response suppression. In fact, we observed the opposite. Binocular facilitation – not suppression – was predominant throughout the period that preceded the onset of perceptual suppression during flash suppression.

Taken together, response suppression because of dichoptic stimulation cannot serve as a ubiquitous mechanism that drives the initiation of rivalry in the form of perceptual suppression. While interocular conflict induced response suppression may still serve as initiator of binocular rivalry under sustained viewing conditions (i.e., simultaneous onset of dichoptic stimuli), it is not causally relevant for the onset of perceptual suppression during BRFS.

At first glance, this finding seems incompatible with the idea that the initial stage of binocular rivalry is due to global destabilization of V1 activity via a reduced signal-to-noise ratio. However, as outlined below, this result is less surprising if interpreted through the lens of visual adaptation. Below, we discuss how our finding rules out interocular conflict induced response suppression as a generalized starting point of perceptual suppression for binocularly competing stimuli, it is still in line with many of the conceptual models that were based on that assumption.

### Monocular adaptation as a mechanistic factor

BRFS is inherently an adaptation paradigm. Monocular adaptation is required to occur for an absolute minimum of 500ms for effective flash suppression to occur.^29^^,^^79^ The main finding of our study is a surprising facilitation of the visual transient following the rivalrous onset of BRFS. This transient BRFS response facilitation can be explained by assuming distinct populations of V1 neurons for each eye and stimulus combination. For example, presenting a horizontally oriented grating to the left eye should maximally excite neurons that prefer the left over the right eye as well as horizontal gratings over vertical gratings. The rivaling stimulus, consisting of a vertically oriented grating presented to the right eye, should maximally excite a non-overlapping population of V1 cells (right eye and vertical grating preferring neurons).

In this scenario, the initial phase of BRFS should maximally adapt (fatigue) one set of neurons. When the second stimulus is presented (flashed) to the other eye, the flashed stimulus will maximally excite a population of neurons that has not been adapted. Indeed, we found that following 800ms of adaptation, V1 responses to the flashed stimulus were nearly identical to those of a simple monocular onset. It is noteworthy that even non-preferred BRFS stimuli evoked neural responses above those of their dioptic counterparts. Yet, this is not surprising since these conditions, too, resemble presentation of a novel stimulus in the first condition and a repeated stimulation with an adapted stimulus in the latter case. In other words, adaptation seems to cause the reduction in responsivity that enables the heightened BRFS transient response.

In this context, it is worth pointing out that this automatic – and thus trivial explanation – for an increased neuronal response to the flashed stimulus (which dominates perception) was already noted by preceding studies on the neural correlates of consciousness that used BRFS. For this reason, these studies omitted the initial transient from their analyses.^28^^,^^29^^,^^173^ Thus, the finding of neuronal correlates of conscious perception reported in these studies is not affected by the mechanistic account of adaptation presented here.

Interestingly, recent work in awake behaving primates demonstrated that there are suppressive interactions between each eye’s signals as early as in the LGN, as well as the input layers of V1.^26^^,^^116^^,^^169^ Similarly, interocular inhibitory activity was also reported by studies of interocular transfer of adaptation.^21^^,^^73^^,^^171^^,^^174^^,^^175^^,^^176^^,^^177^ In other words, left eye and horizontal grating preferring neuronal populations as well as right eye and vertical grating preferring neurons are expected to not only co-exist as two neural populations with nearly orthogonal response properties, but they are also likely to exhibit mutually suppressive interactions.^21^^,^^33^^,^^56^^,^^60^^,^^73^ Assuming this mechanism to exist, flashing the second stimulus not only excites an unadapted population of cells, but also releases their inhibition, thus further explaining the neural facilitation observed in our paradigm.

Lastly, it is worth noting that we observed some form of interocular conflict induced response suppression in the case of BRFS. However, the timing of this interocular conflict induced response suppression is significantly later than previous characterizations,^61^ excluding interocular conflict induced response suppression from having causal relevance for the initiation of perceptual suppression. In other words, the interocular conflict induced response suppression that accompanies BRFS occurs *after* the onset of perceptual suppression that characterizes this paradigm.^79^ It is nonetheless noteworthy that response suppression as a result of dichoptic stimulation is a ubiquitous phenomenon in V1. Recent work demonstrated that a suppressive response to binocular stimuli is not exclusive to (dichoptic) stimuli that cannot be fused, but even occurs for some matching (dioptic) stimuli that do not result in binocular rivalry.^26^

Binocular fusion is arguably one of the main functions of the early visual system, as it gives rise to stereopsis.^178^ Interocular conflict is constantly experienced because of nasal occlusion,^173^ the retinal blind spot,^133^ and non-foveal monocular representations. As such, binocular rivalry is a common occurrence in natural viewing.^179^ Interocular conflict detection and binocular fusion are rapid and automatic processes that occur with every eye movement or blink in natural viewing. Within this context, BRFS is a useful paradigm, as the flashed dichoptic onset mimics interocular discrepancies following a saccade without the confounds of eye movement related activity.

### Adaptation might serve as an alternate trigger for BRFS

If neural response suppression does not induce perceptual suppression during BRFS, what mechanism might be responsible? One interesting clue is that adaptation generally induces reduced V1 neural activity.^102^^,^^116^ Thus, while interocular conflict induced response suppression does not seem to be at play during BRFS, reduced V1 activity could still be a factor.

In contrast to the global effect of interocular conflict induced response suppression, which affects all stimulus-responsive V1 neurons, the reductive effects of adaptation are more specific. Above, we assumed that there are two non-overlapping populations of V1 neurons targeted by a BRFS stimulus, and we assumed that one population is fatigued during the adaptation phase of BRFS. However, we excluded a large fraction of V1 neurons that does not fall into either of these two distinct groups: The majority of V1 neurons receive inputs from and respond to from both eyes.^24^ These binocular neurons also undergo adaptative suppression thanks to interocular transfer of adaptation.^21^^,^^73^^,^^171^^,^^174^^,^^175^^,^^176^^,^^177^ As a result, the adaptive period of BRFS results in rather wide (albeit not global) suppression of V1 responses for the stimulated region of the visual field. As a result, *noise-driven attractor models* could still function within the context of BRFS given that response suppression as a result of dichoptic stimulation was replaced with suppression from adaptation. As such, adaptation might be the mechanism that facilitates the onset of perceptual suppression in BRFS via reduction of visual responses.

We speculate that a similar mechanism might drive the psychophysical dynamics of continuous flash suppression (CFS).^71^^,^^82^^,^^120^^,^^134^^,^^180^^,^^181^ During CFS, Mondrian patches are consistently presented to one eye, thereby continually suppressing the image in the other eye from obtaining perceptual dominance. Thus, BRFS and CFS might operate on a similar mechanism. Each novel Mondrian patch flash captures previously unadapted neuronal populations, and thus continually promotes that eye’s view into perceptual awareness.^82^

It is also intriguing to speculate that we need to look beyond rate codes to fully understand and describe V1’s role for the initiation and maintenance of binocular rivalry. In neural systems, signal flow in the form of increased spiking responses and information flow are fundamentally dissociable.^6^^,^^182^ There are several well-known causally relevant phenomena that occur exclusively at the population level. One example of such phenomena is neuronal synchrony,^13^^,^^183^^,^^184^ while another is systematic changes in correlated noise.^185^^,^^186^ Neither of these causal computations can be detected in analyses of response magnitude alone. Indeed, binocular rivalry has been shown to evoke changes in neuronal coherence in both cats^187^ and primates.^38^ Given what we stated above about the potential of functional differences across cortical layers, it would be intriguing to apply such population-based measures with laminar resolution for the study of binocular rivalry in V1.

### Conclusion: What is the role of adaptation for binocular rivalry more generally?

Despite inclusion of adaptation-related parameters in several newer models of binocular rivalry,^56^^,^^60^ the influence of adaptation on the neurophysiological concomitants of binocular rivalry remains largely unknown.^21^ There are several reasons for an underdeveloped understanding of adaptation’s influence on neural responses during binocular rivalry. First, adaptation-based *opponency models* that lack an *ad hoc* source for random noise demonstrate rivalry oscillations produce regular intervals for perceptual alternations, which is an inaccurate reflection of the binocular rivalry process^75^^,^^188^ (and see Cha & Blake.^189^). *Noise-driven attractor models* improve upon adaptation-based *opponency models* by accounting for random fluctuations in perception,^75^ yet they largely ignore adaptation. Secondarily, neurophysiological work on ongoing binocular rivalry is commonly triggered to the subjects’ report of their perceptual state,^38^^,^^42^ making it difficult to evaluate potential adaption preceding a perceptual transition. Our analyses suggest that further insight can be gained from evaluating the mechanisms of binocular rivalry from the perspective of neuronal tuning preferences and not just the subject’s perceptual state.

### Limitations of the study

One notable caveat with our interpretation is that in the initial documentation of response suppression as a result of dichoptic stimulation in anesthetized cats, the phenomenon was evaluated for several seconds after stimulus onset.^63^^,^^64^^,^^65^^,^^77^^,^^117^ Later investigations in macaques describe suppression 150ms–250ms after stimulus onset,^61^ leaving some ambiguity about the exact timing of this phenomenon.

## STAR★Methods

### Key resources table

**Table:** 

REAGENT or RESOURCE	SOURCE	IDENTIFIER
**Deposited data**		

Macaque V1 recordings during dichoptic stimulation	This paper	https://doi.org/10.5281/zenodo.7949494

**Experimental models: Organisms/strains**		

Bonnet macaque (Macaca radiata)	Wake Forest University, NC, USA	E−48, I-34

**Software and algorithms**		

MATLAB, 2022b	MathWorks	https://mathworks.com/
Custom analysis repository	This paper	https://github.com/BrockMCarlson/BMC_AdaptdcosFigures
Gramm: data visualization toolbox for MATLAB	Pierre Morel, PhD.	https://github.com/piermorel/gramm
JASP: statistical analysis	JASP Team (2023)	https://jasp-stats.org/
NIMH MonkeyLogic: Behavioral Control and Data Acquisition in MATLAB	NIMH	https://monkeylogic.nimh.nih.gov/

**Other**		

Vector Array	NeuroNexus	http://neuronexus.com/products/neural-probes/
U-Probe & V-Probe	Plexon Inc	https://plexon.com/
128 channel neural signal processor	Blackrock Microsystems	http://blackrockmicro.com/
Eye Link II Eye Tracker	SR Research	https://www.sr-research.com/
Cold mirrors	Edmund Optics	https://www.edmundoptics.com/
Data Acquisition Board PCI-6229	National Instruments	http://www.ni.com/en-us.html
Photodiode	OSI Optoelectronics	http://www.osioptoelectronics.com/
Ceramic screws	Thomas Recording	https://www.thomasrecording.com/
Dental Acrylic	Lang Dental Manufacturing	http://www.orthodonticproductsonline.com/buyers-guide/listing/lang-dental-manufacturing-co-inc/
Microdrive	Narishige International USA	https://usa.narishige-group.com/
Plastic Recording Chamber	Crist Instruments	http://www.cristinstrument.com/

### Resource availability

#### Lead contact

Further information and requests for resources should be directed to and will be fulfilled by the corresponding author (alex.maier@vanderbilt.edu).

#### Materials availability

This study did not generate new unique materials. All materials found in the key resources table may be obtained commercially or through open-source repositories.

### Experimental model and study participant details

Animals were trained for a passive fixation task using positive reinforcement. Once both animals performed this task satisfactorily (∼90% trial completion rate), we began electrophysiology recordings. We recorded 14 days (sessions) from two adult monkeys (*Macaca radiata*, one female). 9 recording sessions were performed in subject “E-48” (male, age 9) and 5 recording session were performed in subject “I-34” (female, age 12). During the experimental period, both animals received their daily fluid ration in the form of juice as a reward for the behavioral paradigm. All procedures were approved by the Institutional Animal Care and Use Committee at Vanderbilt University and followed regulations by the National Institutions of Health as well as the Association for the Assessment and Accreditation of Laboratory Animal Care.

### Method details

#### Recording sites and surgical procedures

Recording chambers and headposts were implanted on each monkey in a series of surgeries. Both chambers and headposts were made of custom-designed MRI-compatible plastic (Schmiedt et al., 2014). Sterile surgical conditions were used for all surgeries. Vital signs were monitored continuously and isoflurane anesthesia (1.5-2.0%) was maintained. A craniotomy removed the skull over primary visual cortex's perifoveal visual-field representation. Self-curing denture acrylic (Lang Dental Manufacturing, Wheeling, IL) and transcranial ceramic screws (Thomas Recording, Gießen, Germany) attached the headpost and recording chamber to the skull during surgery. The recording chamber was placed over the position of the craniotomy. Each monkey was given antibiotics and analgesics for postsurgical care.

#### Visual stimulation

Animals sat, head-fixed, in their personalized, custom-designed experimental chairs. Their chairs were placed in front of CRT monitors running at either 60Hz (resolution 1,289 x 1024 pixels) or 85Hz (resolution 1,024 x 768 pixels). Before the start of the experiments, the luminance of the CRT monitors was measured at 17 brightness increments. The CRT monitors were then linearized (“gamma-corrected”) between the minimum and maximum for each gun (red, green, and blue) using a spectroradiometer (Photoresearch, Syracuse, NY). Between the animal and the monitor, a custom-designed dual cold-mirrored stereoscope split the monitor into a left and right eye view (Carmel, Arcaro, Kastner, & Hasson, 2010; Leopold, Maier, & Logothetis, 2003). Images on the right-hand side of the monitor were viewed with the right eye. A black, non-reflective septum divided the two visual fields. 20.5 to 34.5 pixels/^o^ of visual angle (dva) resulted from monitor positioning at 46cm-57cm viewing distance. The monocular visual fields were considered to be aligned when gaze position united across all calibration points on both sides of the monitor (Cox et al., 2019; Dougherty, Cox, Westerberg, & Maier, 2019; Dougherty, Schmid, & Maier, 2019; Westerberg, Cox, Dougherty, & Maier, 2019). Fusion was facilitated by placing an oval aperture at the edge of each half-screen.

#### Behavioral task

Subjects needed to fixate within 1 dva of a fixation cross for the duration of the trial. If the subject fixated for the entire trial (1s-3s), a juice reward was given. No other responses were required. Infrared light-sensitive cameras measured gaze position though infrared-transparent (cold) mirrors.^28^^,^^190^ EyeLink II or SMI Research eye-tracking systems were used to follow the subject’s eye position throughout the experiment. The temporal resolution of the eye-tracking systems was sampled at 500Hz, giving a 2ms resolution.

#### Experimental paradigm

Multi-units were recorded in primary visual cortex (V1) (see data acquisition and multi-unit extraction). For each recording session the general receptive field location was identified using NIMH MonkeyLogic’s receptive field mapper. The subject was required to maintain fixation while the experimenter freely moved a grating across the visual field of each individual eye. The grating patch’s location, size, orientation, spatial frequency, and temporal frequency were adjusted while listening to the excitatory neural activity. The receptive field location was marked on a transparency over the experimenter’s monitor. This information was then used to for a more rigorous follow-up receptive field mapping where recorded multi-units were evaluated by flashing Gabor-filtered random noise patches to various retinotopic positions of the visual field while the subject maintained fixation. The initial receptive field mapping was used to establish a predetermined virtual grid for these noise patches. Full-contrast patches of randomized black and white pixels scaled with eccentricity to accommodate smaller receptive fields near the fovea (.5dva at 1⁰ of eccentricity) and larger receptive fields in peri-foveal areas (up to 3dva at 10⁰ of eccentricity). Mean patch size was .8 dva. Five 200ms stimuli were shown on each trial, with 200ms blank periods interleaved. Retinotopic 3D receptive-field matrices (Cox et al., 2019; Cox et al., 2013) were derived in a reverse-correlation to compute spatial maps of multi-unit responses as a function of visual space.

Once the receptive field of recorded units was determined, their tuning preferences were evaluated. To do so, five 250ms monocular stimuli were interleaved with 250ms blank periods. Sinusoidal gratings were presented that pseudo-randomly varied in phase, spatial frequency, eye, and orientation. Stimulus phase and spatial frequency were matched in each eye. All binocular stimuli were presented with zero disparity (or close to zero disparity given that the monitor was flat rather than curved in the shape of the horopter) between the eyes. After the tuning of select units were evaluated off-line, preferred, and null stimuli were chosen for the day.

Binocular rivalry flash suppression (BRFS) trials started with fixation on a blank screen for 250ms before stimulus presentation (blank periods). Averages of these blank periods were used to compute each multi-unit’s baseline activity. Within the BRFS paradigm both monocular and binocular stimuli were presented. Binocular stimuli were presented as either dioptic controls (same orientation) or dichoptic (orthogonal orientation). On adaptation trials, one eye received monocular stimulation for 800ms before a second stimulus was added in the other eye. We utilized 800ms adaptation because this period of adaptation has been shown to elicit effective BRFS in both humans and macaques (Maier et al., 2007; Wolfe, 1984). We also included a control condition where both stimuli were presented simultaneously without prior adaptation. Presentation of the second stimulus was always in the same receptive field location as the first stimulus (i.e., stimuli occupied corresponding retinal points).

Within BRFS, all grating stimuli were presented at medium-high levels of Michelson contrast (.45-.6). Within each session, the contrast remained constant. Contrast was always balanced (i.e., identical) between the eyes. At higher levels of Michelson contrast, ceiling effects can mask response suppression as a result of dichoptic stimulation, in that dominant eye responses become so prominent that they mask the suppressive influences of the non-dominant eye. A full examination of contrast on interocular conflict induced response suppression can be found in (Cox et al., 2019).

#### Data Acquisition

Linear multielectrode arrays were implanted at the start and removed at the end of each recording session. The electrode contacts along each electrode array were spaced 0.1mm apart, for a total of 24 contacts (UProbe, Plexon Inc., Dallas, TX). These electrode arrays measured extracellular voltage fluctuations against a reference at infinity. A 128-channel Cerebus neural signal processing system (Blackrock Microsystems, Salt Lake City, UT) collected these signals. The Cerebus system amplified, filtered, and digitized the voltage time series. During recordings, subjects sat in an electromagnetic radio frequency shielded booth. A broadband signal (0.3-7.5kHz) was collected at 30kHz and stored for off-line analysis.

To obtain multiunit activity (MUA) from the broadband signal, we low-pass filtered the 30kHz-sampled voltage signals at 5 kHz with a bidirectional Butterworth filter. We then down sampled by a factor of 3, followed by application of a second-order bidirectional Butterworth filter to high-pass filter at 1kHz. We then rectified the resulting data, and down sampled by another factor of 3. To detect impulses (spikes) in the MUA signal, a time-varying threshold enveloped the broadband signal. An impulse was recorded whenever the signal exceeded a pre-set threshold of 2.2 times the noise floor. The threshold was computed after smoothing the signal. Signal smoothing was achieved by convolving the data with a 12s boxcar. Spike waveforms were recovered by extracting ±0.3ms of data from the original signal at each point that the envelope exceeded the threshold. We aligned to the spike waveforms by setting the time-point of each spike to the moment of the signal slope’s maxima within the ±0.3ms window.

Further, several non-neurophysiological signals were collected by the Cerebus signal processing system. Specifically, voltage output of the eye-tracking system was sampled at 1 kHz for eye position information. An analog photodiode signal (OSI Optoelectronics, Montreal, Quebec) for the monitor’s refresh was sampled at 30kHz. Finally, time-stamped event markers were recorded from the behavioral control system (MonkeyLogic). The photodiode signal and the behavioral control system timestamps aligned the extracellular voltage fluctuations to visual and behavioral events.

### Quantification and statistical analysis

Multi-unit responses were taken from the MUA signals described above. Each session was trial-averaged per stimulus condition and then z-score normalized. The z-score normalization was relative to the pre-stimulus baseline responses for each electrode contact. Baseline responses were determined as the mean response for the 50ms leading up to but not including stimulus onset while the subject was fixating on the mean-luminance grey screen. Baseline averages and standard deviations were calculated for all baseline periods over the recording session. The mean and standard error on the mean of condition-specific responses were taken for all contacts within cortex.

Multi-units were classified by their stimulus preferences. To do so, responses were averaged over the 50ms-250ms time-period after stimulus onset for the monocular presentations varying in eye, orientation, contrast, spatial frequency, and phase. Eye and orientation tuning was obtained with MATLAB’s anovan.m function to see if a unit’s response rates varied with both eye-of-origin and orientation. For a unit to be included in all subsequent analyses, it had to be significantly tuned to both eye-of-origin and orientation.

Each unit’s specific orientation preference was determined with a Gaussian curve fitted to the mean response profiles for each sampled stimulus orientation. 18 different gratings of 10⁰ increments from 0⁰ to 180⁰ were used. Preferred orientation was determined by obtaining the maximum of the curve. Null orientation was set as a 90^o^ rotation from the preferred orientation.

We used characteristics of local field potentials (LFP), current source density (CSD), power spectral density (PSD), and multi-unit activity (MUA) to determine cortical boundaries,^6^^,^^24^^,^^61^^,^^116^ see^58^ for histological verification. Additionally, we ensured receptive fields were aligned across cortical depths. Aligned receptive fields indicate that our acute electrode penetrations were orthogonal to the cortical surface, and aid in the determination of V1 upper and lower boundaries. Out of 360 recordings (24 contacts on each laminar probe, used across 15 sessions), 219 were determined to be within V1 cortical boundaries (142 in “E” and 77 in “I”). Of these 219 V1 multi-units, 128 did not show significant tuning to both eye and orientation and were removed from the analysis. 91 V1 multi-units demonstrated significant tuning to both eye and orientation (72 from “E”, and 19 from “I”), and were used in all subsequent analyses. Table 2 shows the subject and sample information.

**Table 2:** Subject and sample information

Subject	Sessions	Tuned units	Untuned units	Total
E48	9	72	70	142
I34	5	19	58	77
Total	14	91	128	219

Tuned units must be tuned to both eye and orientation to be included in our analysis.

Statistical analyses were performed via MATLAB or using the open-source statistics JASP software package (JASP Team (2022). Version 0.16.3). When assumptions of normality were violated, non-parametric tests were used. Our data analysis primarily comprised of pairwise comparisons, so mean MUA responses were evaluated via Wilcoxon signed-rank tests.

## Data Availability

• Event-triggered multi-unit-activity is published on Zenodo, and it is publicly available as of the date of publication. The DOI is listed in the key resources table. • All original code is published on GitHub in the repository BMC_AdaptdcosFigures. Accession numbers are listed in the key resources table. • Any additional information required to reanalyze the data reported in this paper is available from the lead contact upon request.
